# Moderate and vigorous aerobic exercise enhances inhibitory control, but not working memory or cognitive flexibility, up to the second ventilatory threshold: a randomized crossover trial

**DOI:** 10.1016/j.clinsp.2026.100896

**Published:** 2026-02-24

**Authors:** Marcelo Hiro Akiyoshi Ichige, Paulo Roberto Santos-Silva, Julia Maria D’Andrea Greve

**Affiliations:** Laboratorio de Estudos do Movimento, Instituto de Ortopedia e Traumatologia do Hospital das Clínicas, Faculdade de Medicina, Universidade de São Paulo (HCFMUSP), São Paulo, SP, Brazil

**Keywords:** Executive functions, Physical exercise, Physical activity, Inhibitory control, Working memory, Cognitive flexibility

## Abstract

**Introduction:**

The effects of acute physical exercise on executive functions remain controversial, partly due to inconsistencies in how exercise intensity is measured. This study investigated the impact of moderate and vigorous aerobic exercise on three core components of executive function: inhibitory control, working memory, and cognitive flexibility. Exercise intensity was rigorously defined using a combination of heart rate reserve, ventilatory thresholds, and rate of perceived exertion.

**Method:**

Twenty-six physically active adults completed three separate sessions in a randomized order: Rest (REST), Moderate-intensity exercise (MOD), and Vigorous-Intensity exercise (VIG). Cognitive performance was assessed during each condition using the Go/No-Go test (inhibitory control), the 2-back test (working memory), and the Multitasking test (cognitive flexibility).

**Results:**

Inhibitory control improved significantly during exercise. Response Times (RT) on the Go/No-Go test were reduced in both MOD (*p* = 0.038) and VIG (*p* = 0.003) compared with REST, without changes in accuracy. In contrast, working memory and cognitive flexibility showed no significant differences across conditions.

**Conclusions:**

Acute moderate and vigorous aerobic exercise, up to the second ventilatory threshold, selectively enhances inhibitory control by reducing RT, with no detectable effects on working memory or cognitive flexibility. By integrating multiple physiological and perceptual markers of intensity, this study provides a rigorous evaluation of the acute cognitive effects of aerobic exercise.

## Highlights

• Executive functions components behave differently during aerobic exercise.
• Inhibitory control improves during moderate and vigorous exercise.
• No effect of moderate or vigorous exercise on working memory or cognitive flexibility.

## Introduction

For decades, physical activity has been recognized as an independent factor in reducing all-cause mortality and the risk of cardiovascular disease.^1^ Beyond its impact on general health, substantial evidence indicates that regular physical activity enhances cognitive functions and may reduce the risk of dementia and Alzheimer’s disease.^2^, ^3^, ^4^ Acute physical activity has also been shown to benefit cognition when assessed immediately after exercise^5^ however, its effects during exercise remain less explored and more controversial, varying according to exercise intensity and the specific cognitive domain assessed.^6^^,^^7^

Within these domains, executive functions ‒ or cognitive control ‒ are of particular relevance due to their hierarchical role in regulating other cognitive processes and their importance for daily functioning. Executive functions comprise a set of mental processes required for tasks that demand concentration, planning, and actions beyond automatic responses. Higher executive function performance has been linked to greater academic, professional, and social success,^8^ as well as enhanced sports performance among athletes.^9^^,^^10^

Current consensus identifies three main components of executive function: inhibitory control, working memory, and cognitive flexibility.^8^^,^^11^ A recent meta-review found no overall change in cognitive performance during physical activity when analyzed broadly.^5^ However, when executive functions were examined separately, improvements were noted across all intensities and assessment timings.^6^ Evidence suggests that these benefits are most pronounced at moderate intensities, with diminished effects at lighter or more vigorous intensities, supporting a potential *U*- or *J*-shaped relationship. Reviews focusing specifically on executive function components highlight that inhibitory control has been the most investigated and generally follows this *J*-shaped trend ‒ improving slightly at moderate intensity but declining at vigorous levels. In contrast, working memory and cognitive flexibility tend to remain stable during moderate exercise and may deteriorate when intensity increases.^7^

In spite of the trends suggested by current literature, the limited number of studies and their conflicting findings complicate our understanding of these effects. A major source of discrepancy lies in the assessment of exercise intensity. Many studies rely on self-selected intensities, fixed loads that ignore individual fitness levels, or simple percentages of maximum heart rate or VO2.^12^ These approaches have clear limitations, as they do not account for resting values and show weak correlations with metabolic markers of intensity. More individualized methods, such as percentage of heart rate reserve or VO2 reserve, are rarely applied and still provide only partial accuracy. A more robust alternative is the use of ventilatory thresholds, which better reflect the physiological demands of exercise. In addition, studies incorporating internal load ‒ strongly influenced by mood, environment, sleep, diet, and fitness level ‒ remain scarce.^12^ These methodological challenges are not exclusive to cognition research but extend broadly across exercise science.^13^ Furthermore, most existing studies focus on only one or two components of executive function, limiting conclusions about their differential responses to exercise.

The objective of this study is to evaluate the three core components of executive function during moderate and vigorous ‒ though not severe ‒ aerobic exercise, using a robust approach that integrates cardiac (heart rate reserve), metabolic (ventilatory thresholds), and psychological (rate of perceived exertion) parameters. Based on the proposed *U*-shaped relationship between cognitive performance and exercise intensity, the authors hypothesize that carefully and individually defined intensities within this range do not impair executive function. Furthermore, the authors propose that a more precise definition of exercise intensity, combined with separate analyses of inhibitory control, working memory, and cognitive flexibility, will provide clearer insights into the existing evidence suggesting differential sensitivity of executive functions components to exercise. To address these gaps, this study compares executive function performance across three conditions: rest, moderate exercise, and vigorous exercise, applying rigorous and multidimensional criteria to determine intensity.

## Methods

### Study design and participants

Twenty-six physically active adults, recruited among students and staff of Hospital das Clínicas da Faculdade de Medicina da Universidade de São Paulo, participated in this study. They engaged in at least 150 min of moderate exercise or 75 min of vigorous exercise each week, but none were professional athletes. All participants had normal or corrected-to-normal vision and did not have any cardiometabolic or musculoskeletal conditions that might limit their ability to perform physical exercise. There were no losses to follow-up or dropouts. This study was a randomized, crossover trial and is reported in accordance with the Consolidated Standards of Reporting Trials (CONSORT) guidelines. All participants signed an informed consent form. The study was approved by the Ethics Committee of the Hospital das Clínicas da Faculdade de Medicina da Universidade de São Paulo (83554524.6.0000.0068). The research was conducted in accordance with the Declaration of Helsinki.

### Cardiopulmonary exercise test

On the day of the experiments, participants were instructed to get adequate sleep the night before and to avoid caffeine or other stimulants. On the first day, they underwent a pre-participation evaluation that included a medical history review and physical examination to ensure their safety for vigorous physical activity. They then performed an incremental cardiopulmonary exercise test on a cycle ergometer (Wattbike Air, WattBike, West Bridgford, UK). Continuous monitoring included a 13-lead electrocardiogram (HeartWare, Ergo 13, Belo Horizonte, Brazil), blood pressure measurements, and assessments of pulmonary ventilation (VE), oxygen consumption (VO2), end-expiratory oxygen pressure (PETO2), CO2 production (VCO2), and end-expiratory carbon dioxide pressure (PETCO2) using a computerized gas exchange analysis system (CPX/Ultima, Medical Graphics, MN, USA).

Participants were encouraged to exert maximum effort, and peak VO2 and peak heart rate were defined based on meeting at least two of the following three criteria:

1. A lack of VO2 increase despite increased exercise intensity (a variation <2.1 mL/kg/min).
2. A heart rate exceeding 90 % of the maximum predicted for their age, as calculated using the Tanaka equation [208 − (0.7 × age)].
3. A respiratory quotient (RQ, calculated as VCO2/VO2) of 1.10 or higher.

The first (VT1) and second (VT2) ventilatory thresholds were identified through an analysis of VE, VE/VO2, VE/VCO2, PETO2, and PETCO2, as described in previous studies.^14^

### Exercise sessions

On a different day, participants were assigned to one of three exercise intensity conditions in a randomized order: Rest (REST), Moderate-intensity exercise (MOD), and Vigorous-intensity exercise (VIG). Each session was separated by at least one week to minimize learning effects. The time between the cardiopulmonary exercise test and the last experimental session did not exceed eight weeks to avoid significant changes in VO2 or ventilatory thresholds.

The experiments were conducted on a cycle ergometer (Max-KS5, Kikos Fitness, São Paulo, Brazil). Participants were monitored using a heart rate monitor (Polar H9, Polar Electro Oy, Kempele, Finland). A table displaying the Borg scale of perceived exertion (ranging from 6 to 20) was visible during each session. Exercise intensities were defined based on the American College of Sports Medicine (ACSM) recommendations^15^ and utilized ventilatory thresholds to determine transitions between light and moderate exercise and from vigorous to severe exercise:

– Rest Session: Participants remained seated on the cycle ergometer without pedaling. Cognitive tests began after five minutes of rest.
– Moderate Exercise Session: This session included a five-minute warm-up with a progressively self-selected load, followed by exercise at an intensity between VT1 and 60 % of heart rate reserve. Participants reported a rate of perceived exertion between 12 and 13 on the Borg scale.
– Vigorous Exercise Session: This session also began with a five-minute warm-up with a progressively self-selected load, followed by exercise at an intensity between 70 % of heart rate reserve and VT2. Participants reported a rate of perceived exertion between 15 and 17 on the Borg scale.

Participants were instructed to maintain their heart rate within the designated range while aiming to closely match the indicated rate of perceived exertion. If a participant's heart rate fell outside this range, the researcher made slight adjustments to the ergometer load to ensure proper test execution without interruption.

### Cognitive assessment

The cognitive assessment included the Multitasking test for cognitive flexibility,^16^ the 2-back test for working memory, and the Go/No-Go test for assessing inhibitory control.^8^ These tests were administered in this specific order during each exercise session, using PsyToolkit software^17^^,^^18^ on a 15.6-inch screen with a 60 Hz refresh rate (Vivobook 15, ASUS, Taipei, Taiwan), positioned about 80 cm from the participant. A portable QWERTY keyboard (KA-1118, Shenzhen Hongxin Weichuang Technology, Shenzhen, China) was used for responses.

All participants were introduced to the cognitive tests on the day of the cardiopulmonary exercise test. During the exercise sessions, the test instructions were reiterated, and a brief training session was conducted. Participants were encouraged to perform their best in terms of both accuracy and Response Time (RT) and were instructed not to practice the tests outside of the designated sessions. Each session began after a five-minute warm-up in the exercise sessions or a five-minute waiting period in the REST session, with all sessions lasting no longer than twenty minutes.

### Multitasking test

**Fig. 1:**
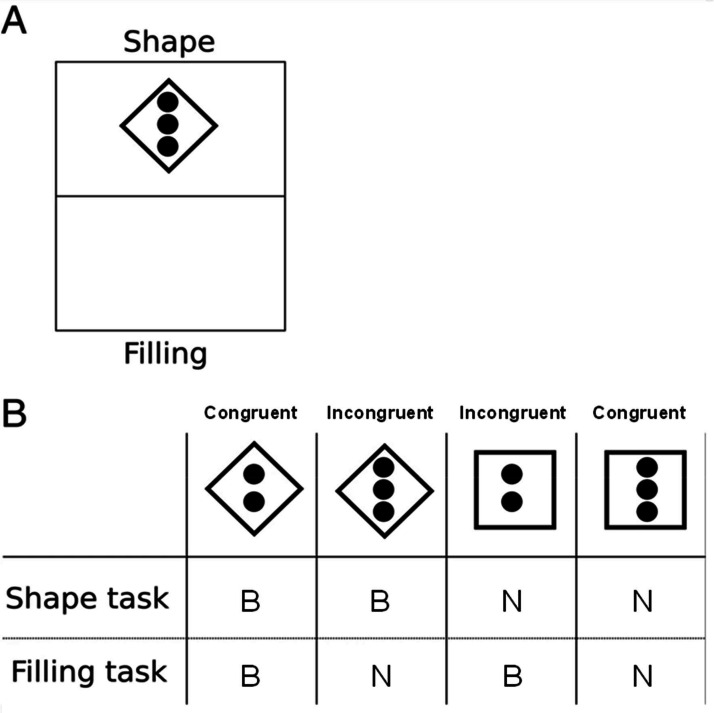
Schematic representation of the Multitasking test. (A) Each trial is composed by a horizontally divided rectangle where the top half corresponds to the shape task and the bottom half to the filling task. The participant should respond according to the location of the stimulus. In this example, the stimulus is located in the top half and the participant should respond according to the diamond shape. (B) Correct responses for each stimulus in each task type. For the shape task, the participant should respond with B for the diamond and N for the square, ignoring the dots inside the figure. In the filling task, the participant should respond with B for two dots and N for three dots, ignoring the external shape of the figure. Adapted from Stoet et al.^16^ (licensed under CC BY 2.0).

The Multitasking Test evaluates the ability to switch between tasks quickly. A rectangular area measuring 6 × 6.5 cm was utilized, with the top half labeled “shape” and the bottom half labeled “filling” (Fig. 1A). Four different stimuli (Fig. 1B) were used, which combined either a diamond or square shape with a filling of two or three circles. For the shape task (top half), participants pressed the 'B' key for a diamond and the 'N' key for a square, regardless of the filling. For the filling task (bottom half), participants pressed the 'B' key for two circles and the 'N' key for three circles, regardless of the shape.

**Fig. 2:**
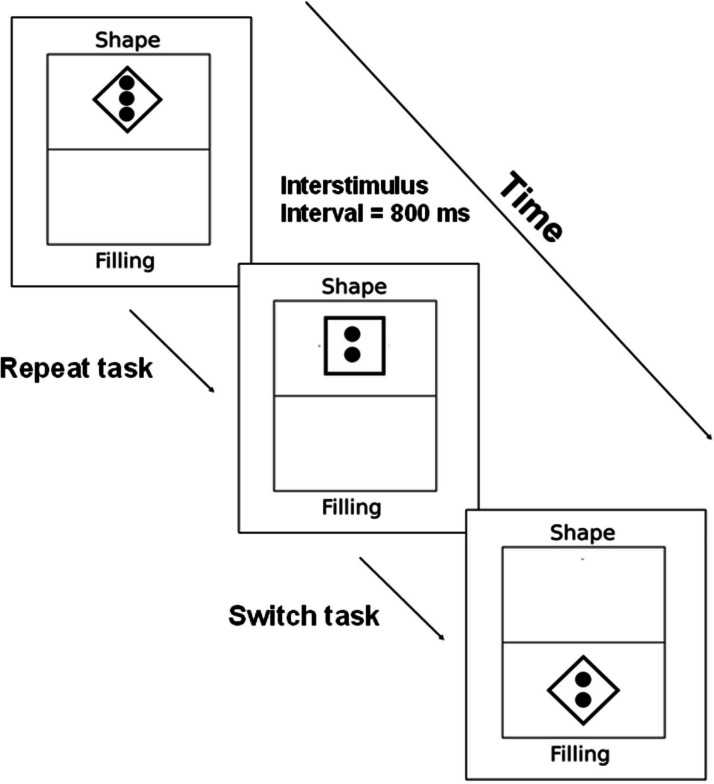
Task flow of the Multitasking test. One stimulus was presented at a time, located in the top or bottom half of the rectangle. The stimulus disappeared after the participant provided a response or after four seconds. The interval between stimuli was 800 ms. Tasks preceded by the same type of task (shape or filling) were called Repeat. Tasks preceded by the alternative task were called Switch.

The stimuli appeared yellow on a black background, with participants having up to four seconds to respond to each stimulus. The stimulus changed after a response or when the time limit was reached. An 800 ms pause occurred between each stimulus (see Fig. 2). If a participant made an error, a message indicating the type of error (wrong key or time expired) was displayed for one second, followed by an image reinforcing the instructions for five seconds, and then a 500 ms pause before resuming.

In this test, half of the stimuli were congruent (where the shape and filling corresponded to the same key) and half were incongruent (where they corresponded to different keys). This required participants to determine which information was relevant based on the stimulus location (top or bottom half). Each experimental session included three training blocks: the first focused solely on the shape task (five stimuli), the second on the filling task (five stimuli), and the third presented both tasks randomly (ten stimuli). Following the training, the data acquisition block began, consisting of 80 stimuli ‒ 40 from each task ‒ presented randomly. Stimuli preceded by the same task were categorized as “Repeat”, while those preceded by a different task were categorized as “Switch”. The main outcomes analyzed included the percentage of errors, the mean RT of correct responses in Switch tasks, and the difference between the mean RT in Switch and Repeat tasks, referred to as “switch cost”.

### 2-Back test

**Fig. 3:**
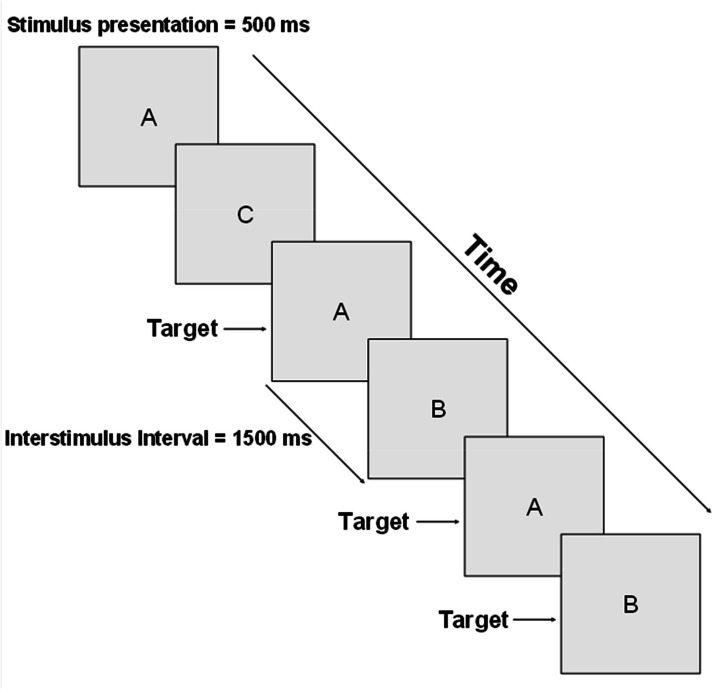
Task flow of the 2-back test. The stimuli (letters) were presented one at a time for 500 ms, followed by a black screen for 1500 ms, resulting in 2000 ms for the participant to respond (M key) in case of a target or to omit their response in case of a non-target. Target stimuli were those that were the same as the second-to-last stimulus (2-back). Target stimuli could appear sequentially (e.g., A-B-A-B).

The 2-back test measures the ability to store, update, and compare stimuli. Participants viewed a pseudorandomized sequence of letters, one at a time, among a group of fifteen (A, B, C, D, E, H, I, K, L, M, O, P, R, S, T), displayed in uppercase black Arial font inside a yellow square on a black background. They must press the M key when the current stimulus matches the one from two steps earlier (e.g., A-B-A). Each stimulus is shown for 500 ms, followed by a 1500 ms black screen, giving participants 2 s to respond (see Fig. 3).

Each session included a training block of fifteen stimuli and a data acquisition block of sixty stimuli. Responses were categorized as matches, misses (failure to press on a target), and false alarms (pressing on a non-target). The analysis focused on RT for matches, the percentage of miss errors, and the percentage of false alarm errors.

### Go/No-go test

The go/no-go test evaluates the ability to suppress impulsive responses. Participants were presented with two types of stimuli: a go stimulus, represented by a green ellipse measuring 6 × 2 cm with the word “GO” in black inside it, and a no-go stimulus, represented by a red ellipse of the same dimensions with the text “NO-GO”. These stimuli were displayed against a black background.

**Fig. 4:**
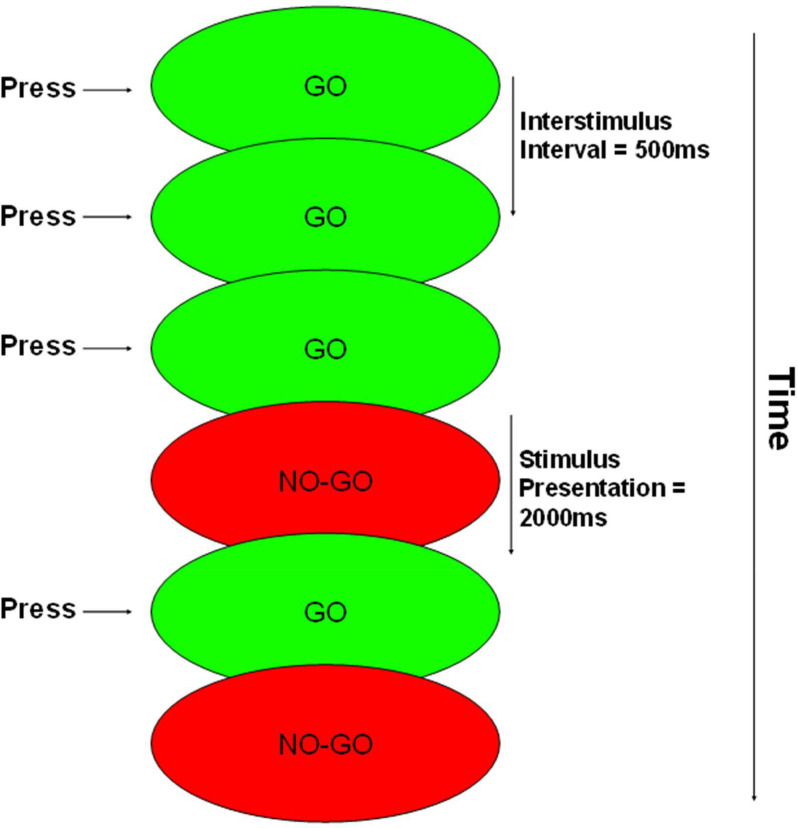
Task flow of the Go/No-Go test. Stimuli were presented one at a time for 2000 ms or until the participant provided a response (space key). The interval between stimuli was 500 ms. Correct responses occurred when the participant provided a response in <2000 ms to Go stimuli or omitted their response to No-Go stimuli. Commission errors occurred when the participant responded to a No-Go stimulus. Omission errors occurred when the participant failed to respond to a Go stimulus.

Participants were instructed to press the space bar whenever the go stimulus appeared and to refrain from pressing it in the presence of the no-go stimulus. Each stimulus was presented for 2000 milliseconds or until the participant pressed the space key, with a 500-millisecond interval between stimuli (see Fig. 4). If participants made an error, a feedback message was displayed for 2 s.

The test included a total of 100 trials, consisting of 80 go stimuli and 20 no-go stimuli presented in a random order. RT and the percentage of commission errors (errors made on no-go trials) were analyzed. There were no omission errors recorded for any participant in any exercise condition.

### Data and statistical analysis

RT outliers exceeding ±3 standard deviations from each participant’s mean within each condition were excluded. Dependent variables were tested for sphericity using Mauchly’s test; when violated, the Greenhouse-Geisser correction was applied. RT was analyzed using repeated-measures ANOVA, and error rates ( %) were analyzed with the Friedman test, with exercise intensity (three levels) as the within-subject factor for each dependent variable. Effect sizes were reported as partial eta-squared (ANOVA) and Kendall’s W (Friedman). Significant effects were followed by pairwise comparisons between exercise intensities using paired *t*-tests with Holm-Bonferroni correction; Cohen’s d was reported as the effect size. A significance threshold of *p* < 0.05 was adopted.

For nonsignificant results, Bayesian repeated-measures ANOVA was additionally conducted, and Bayes factors (BF₀₁) were reported. Data are expressed as mean ± standard deviation. All analyses and figures were performed using JASP^19^ software.

## Results

Table 1 presents the demographic and cardiopulmonary exercise test data for the participants. All our participants were university graduates or post-graduates, with a relatively homogenous age distribution and high variability in weekly exercise distribution.

**Table 1:** Participant demographics and cardiopulmonary exercise test results. Data are presented as mean ± standard deviation.

Number of participants	26 (11 female)
Age (years)	30.5 ± 3.2
Weight (kg)	73.2 ± 13.5
Height (cm)	169.5 ± 9.9
Years of education (years)	19.8 ± 1.7
Weekly exercise (minutes)	363.8 ± 182.2
Peak heart rate (beats/min)	182.5 ± 9.1
Peak VO2 (mL/kg/min)	38.9 ± 5.7
Resting heart rate (beats/min)	74.2 ± 9.8
Heart rate at VT1	130.6 ± 8.5
Heart rate at VT2	161.7 ± 8.5

### Cognitive performance across executive function components

**Fig. 5:**
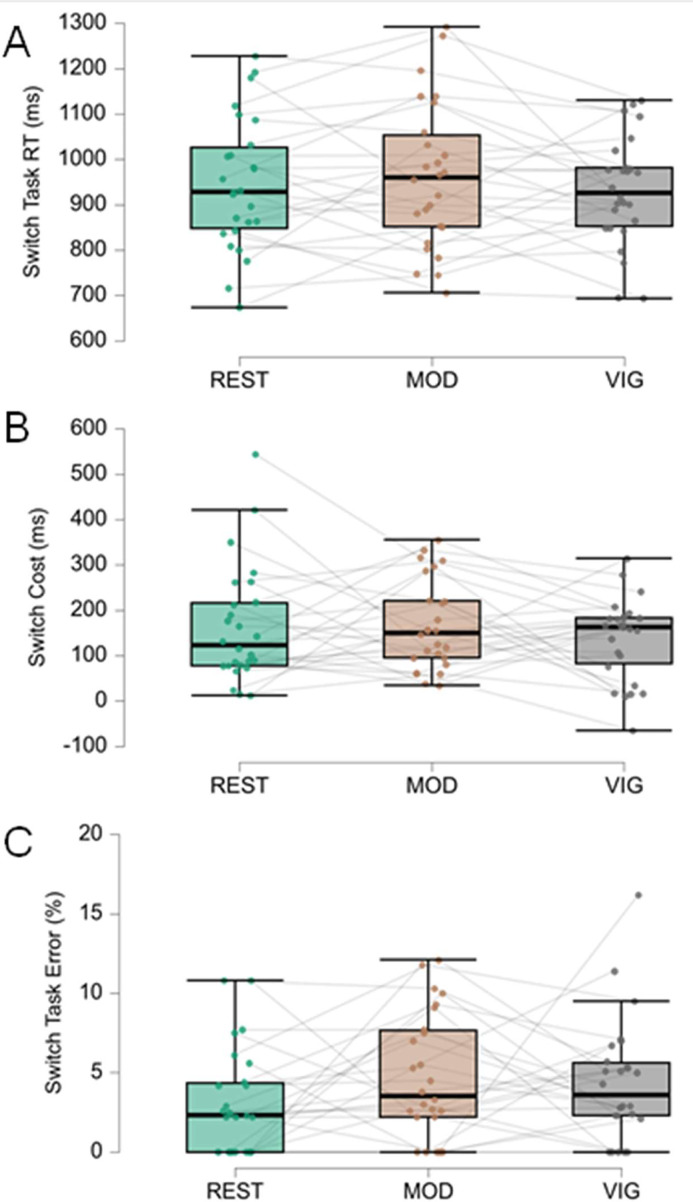
Results of the Multitasking test. (A) Response Time (RT) in ms in Switch tasks in REST, MOD, and VIG exercise conditions. (B) Switch cost in ms in the three exercise conditions. (C) Percentage of errors in Switch tasks in the three exercise conditions. Each interconnected dot represents one participant. The horizontal line within the box represents the median. Upper and lower limits of the box represent, respectively, first and third quartiles. Whiskers represent maximum and minimum values within 1.5 times the interquartile range.

**Fig. 6:**
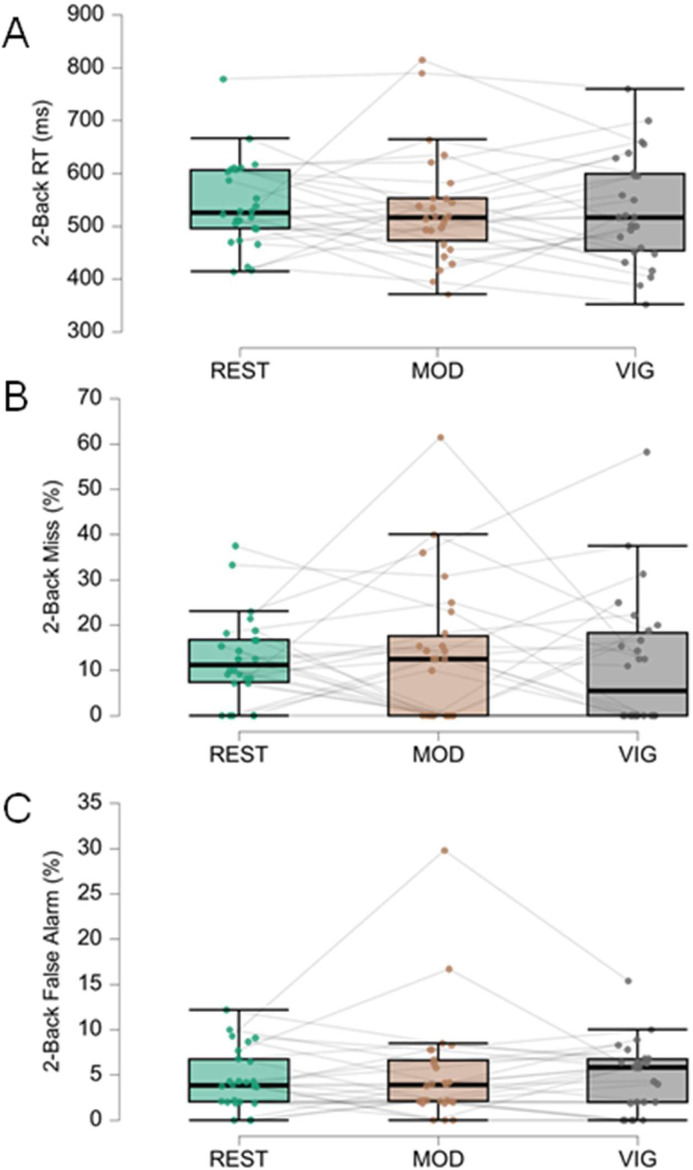
Results of the 2-back test. (A) Response Time (RT) in ms of Match responses (participant correctly responded to a target) in REST, MOD, and VIG exercise conditions. (B) Percentage of Miss errors (participant failed to respond to a target) in the three exercise conditions. (C) Percentage of False-alarm errors (participant responded to a non-target) in the three exercise conditions. Each interconnected dot represents one participant. The horizontal line within the box represents the median. Upper and lower limits of the box represent, respectively, first and third quartiles. Whiskers represent maximum and minimum values within 1.5 times the interquartile range.

**Fig. 7:**
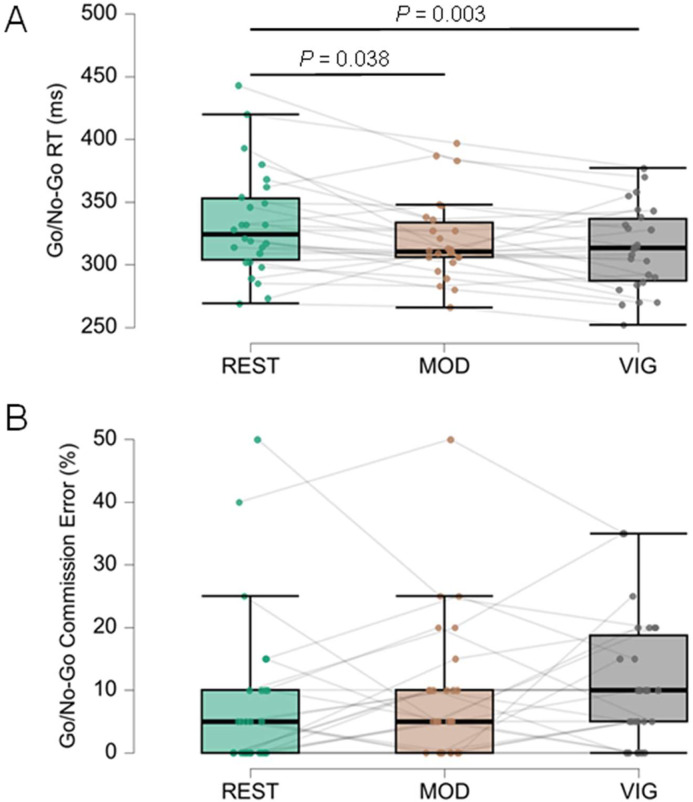
Results of the Go/No-Go test. (A) Response Time (RT) in ms of correct responses to the Go stimulus in REST, MOD, and VIG exercise conditions. (B) Percentage of Commission errors (participant responded to a No-Go stimulus) in the three exercise conditions. Each interconnected dot represents one participant. The horizontal line within the box represents the median. Upper and lower limits of the box represent, respectively, first and third quartiles. Whiskers represent maximum and minimum values within 1.5 times the interquartile range. Significant differences were highlighted with a horizontal line and the p-value from the post-hoc analysis.

Fig. 5, Fig. 6, Fig. 7 present the outcomes of the three cognitive tasks assessing executive function components during rest, Moderate (MOD), and Vigorous (VIG) exercise.

Cognitive flexibility (Multitasking test) showed no significant differences across intensities for RT in switch tasks [REST = 946 ± 145 ms; MOD = 963 ± 161 ms; VIG = 931 ± 119 ms; F(2,25) = 0.77, *p* = 0.470, ηp²=0.03], switch cost [REST = 164 ± 129 ms; MOD = 167 ± 99 ms; VIG = 137 ± 90 ms; F(2,25) = 0.75, *p* = 0.479, ηp² = 0.029], or percentage of errors [REST = 3.1 %% ± 3.28 %; MOD = 4.71 % ± 3.96 %; VIG = 4.39 % ± 3.81 %; χ²(2) = 3.62, *p* = 0.163, Kendall’s *W* = 0.07]. Bayesian analysis indicated moderate evidence for the null hypothesis for RT (BF₀₁ = 5.0) and switch cost (BF₀₁ = 4.8), and weak evidence for errors (BF₀₁ = 2.4).

Working memory (2-back test) also showed no significant differences for RT [REST = 541±83 ms; MOD = 533±106 ms; VIG = 532±103 ms; F(2,25) = 0.149, *p* = 0.862, ηp²=0.006], Miss errors [REST = 12.4 % ± 9.5 %; MOD = 13.1 % ± 15.6 %; VIG = 11.4 % ± 14.8 %; χ²(2) = 0.16, *p* = 0.924, Kendall’s *W* = 0.003], or False-alarm errors [REST = 4.6 % ± 3.3 %; MOD = 5.2 % ± 6.2 %; VIG = 5.0 % ± 3.7 %; χ²(2) = 0.14, *p* = 0.932, Kendall’s *W* = 0.003]. Bayesian analysis provided moderate evidence for the null hypothesis (BF₀₁ = 7.9–6.9).

Inhibitory control (Go/No-Go test) differed by exercise intensity for RT [REST = 333 ± 43 ms; MOD = 320±32 ms; VIG = 313 ± 33 ms; F(2,25) = 8.516, *p* < 0.001, ηp² = 0.254]. Post-hoc tests revealed faster RTs with a moderate effect size for VIG vs. REST (*p* = 0.003, *d* = 0.543) and small-to-moderate effect for MOD vs. REST (*p* = 0.038, *d* = 0.352), with no difference between MOD and VIG (*p* = 0.096, *d* = 0.191). No significant differences were observed for commission errors [REST = 8.1 % ± 12.7 %; MOD = 9.4 % ± 11.3 %; VIG = 11.0 %±10.3 %; χ^2^(2) = 3.455, *p* = 0.178, Kendall’s *W* = 0.066], with moderate Bayesian evidence for the null (BF₀₁ = 3.8).

## Discussion

Our study revealed a significant improvement in inhibitory control, as evidenced by decreased RT in the Go/No-Go test during both moderate and vigorous exercise up to VT2, while accuracy remained unchanged. In contrast, working memory and cognitive flexibility, assessed through the 2-back and multitasking tests, did not differ across exercise conditions. This study is among the first to employ a method of measuring exercise intensity that simultaneously includes cardiac (heart rate reserve), metabolic (ventilatory thresholds), and perceptual (Borg) criteria to evaluate all three core components of executive functions. Additionally, our sample size was larger than average for studies in this field.

The absence of significant changes in cognitive flexibility during moderate exercise is consistent with previous research.^20^^,^^21^ Findings at vigorous intensities are more variable and often depend on task type and duration. For instance, studies using the Wisconsin Card Sorting Test reported impaired performance during 40–45 min of vigorous exercise.^21^^,^^22^ Such prolonged protocols may elevate internal load beyond the calculated intensity, potentially explaining the discrepancy with our results. Other studies using task-switching or Stroop paradigms have reported declines in accuracy,^20^ sometimes linked to less precise intensity definitions (e.g., fixed percentages of maximal power or VO2max). Methodological differences, including task duration, participant fitness, and non-randomized exercise order, likely contribute to conflicting findings.

Research on working memory during exercise is less extensive but generally supports our results. Prior studies have reported no changes in RT or accuracy during moderate exercise.^23^^,^^24^ At vigorous intensities, some evidence suggests declines only with prolonged exercise or repeated testing.^25^ These effects may reflect a combination of physical and mental fatigue. Notably, protocols that reported impairments often used small, male-only samples, different task modalities (e.g., spatial working memory), or graded intensities without randomization ‒ factors that limit comparability with our findings.^24^

Our findings on inhibitory control align with most prior studies,^7^ particularly at moderate intensities. Discrepancies across the literature may stem from differences in task selection. While Go/No-Go tasks often reveal improvements, studies assessing related constructs such as interference control or selective attention have yielded mixed results.^26^^,^^27^ Protocols employing lower intensities (e.g., 50 % VO2max, close to or below VT1) or methodological limitations such as non-randomized session order also reduce comparability.^24^ The literature on vigorous exercise is especially heterogeneous. While some studies report improvements, others report no effects, and some even show trends toward slower RTs.^26^^,^^28^ Declines in accuracy typically appear at severe intensities, likely at or above VT2.^24^^,^^26^^,^^28^^,^^29^ In addition, differences in participants' demographics, type of cognitive tasks and absence of randomization in exercise intensity order^24^^,^^26^ might also contribute to the conflicting results.

The difference the authors have observed between the behavior of inhibitory control during exercise, when compared to working memory and cognitive flexibility, has been described before.^7^ This finding could be related to the former's potential role as a foundational or common factor within executive functions.^30^ Developmental studies have found inhibitory control to be the first component of executive functions to mature^31^ and it has been proposed that working memory and cognitive flexibility build upon inhibitory control.^8^ It is possible that the higher need of resources from working memory and cognitive flexibility limits room for their improvement during exercise. A few studies have investigated mechanistic explanations for the behavior of cognitive functions during exercise,^32^, ^33^, ^34^ but this area remains speculative and warrants future studies.

Our study was one of the few that assessed exercise intensity using heart rate reserve, ventilatory thresholds, and internal load criteria simultaneously. By carefully distinguishing vigorous from severe exercise, our study demonstrates that executive function is preserved up to VT2. Supporting this, impairments in accuracy are typically observed only at severe intensities, likely exceeding VT2.^24^^,^^26^^,^^28^^,^^32^^,^^35^ This challenges overly simplistic reliance on percentages of VO2max or HRmax to classify exercise intensity and highlights the value of ventilatory thresholds, which better reflect metabolic stress.^12^^,^^13^^,^^36^^,^^37^ Methods based on percentage of maximal anchors (e.g., maximal heart rate, VO2max and peak work rate) often result in arbitrary divisions for exercise intensity ranges, limiting their correlation with markers of homeostatic changes and leading to greater interindividual variation.^13^ On the other hand, there is considerable evidence that VT1 indicates the transition between light and moderate intensity exercise.^13^ Also, although identifying VT2 carries some inter-observer variability,^38^ it remains a more robust criterion than conventional markers and aligns with its proposed role as an indicator of maximal metabolic steady state.^36^^,^^37^ Our study did not evaluate intensities above VT2 as it would result in non-steady-state exercise, requiring different protocols such as intermittent exercise or shorter cognitive tasks. While established as a metabolic threshold, future studies are necessary to determine if VT2 also acts as a cognitive threshold.

The authors recognize several limitations in our study. First, repeated task exposure, inherent to the repeated measures method, may have induced learning effects. To mitigate this, the authors separated sessions by at least one week and randomized exercise intensity order. Second, the high education level of our sample raises the possibility of ceiling effects in accuracy measures that may have limited the sensitivity to detect exercise-induced changes in some tasks. Nevertheless, RT performance was less likely to be constrained, and participants’ baseline results were consistent with published normative data from larger studies.^16^^,^^39^^,^^40^ Third, the relatively short exercise duration (< 20-minutes) limits extrapolation to longer sessions, where fatigue effects are more pronounced.^25^ Finally, the fixed order of cognitive tasks may have influenced outcomes. As previously discussed, inhibitory control is demanded by working memory and cognitive flexibility tasks. The authors cannot exclude the influence of practice effects of the multitasking and the 2-back tests on the Go/No-Go test. On the other hand, even in the relatively short duration of each exercise session, the authors cannot exclude the possibility of some level of mental or physical fatigue within the session that could have particularly affected our findings regarding inhibitory control, which was always assessed last.

## Conclusions

Our study shows that during moderate to vigorous aerobic exercise (between VT1 and VT2), response time improves without a change in the accuracy of inhibitory control. The other components of executive function, working memory, and cognitive flexibility, were unaffected by the exercise intensities used.

## Funding

This project received no funding from any public, commercial, or non-profit agency.

## CRediT authorship contribution statement

**Marcelo Hiro Akiyoshi Ichige:** Conceptualization, Methodology, Software, Validation, Formal analysis, Investigation, Data curation, Writing – original draft, Visualization. **Paulo Roberto Santos-Silva:** Conceptualization, Methodology, Investigation. **Julia Maria D’Andrea Greve:** Conceptualization, Methodology, Validation, Resources, Writing – review & editing, Supervision.

## Declaration of competing interest

The authors declare no conflicts of interest.

## Data Availability

The data that support the findings of this study are available from the corresponding author upon reasonable request.
